# Impact of Cataract Surgery on Functional Balance Skills of Adults

**DOI:** 10.4274/tjo.galenos.2019.70104

**Published:** 2019-10-24

**Authors:** Fulya Duman, Zeynep Kılıç, Emel Ece Özcan-Ekşi

**Affiliations:** 1 University of Health Sciences, Antalya Health Practice and Research Center, Department of Ophthalmology, Antalya, Turkey; 2 Yıldırım Beyazıt University, Atatürk Training and Research Hospital, Physical Medicine and Rehabilitation Clinic, Ankara, Turkey; 3 Bahçeşehir University Faculty of Medicine, Department of Physical Medicine and Rehabilitation, İstanbul, Turkey

**Keywords:** Vision, cataract, balance, phacoemulsification, falls

## Abstract

**Objectives:**

To investigate the impact of phacoemulsification surgery and intraocular lens implantation on the functional balance skills of adults.

**Materials and Methods:**

This prospective study included patients with cataract who were recommended phacoemulsification surgery and intraocular lens implantation between May and October 2016. The Berg Balance Scale and Tinetti Gait and Balance Test were performed by a physical therapy specialist before and 1 month after surgery. Patients were analyzed in terms of age, visual acuity, and balance. Balance scores before and after cataract surgery were compared. We also compared patients with high (≤2 LogMAR) and low (>2 LogMAR) visual acuity. P values below 0.05 were accepted as statistically significant.

**Results:**

Fifty-one patients (27 female and 24 male, mean age 66.96 years) were included in the study. One month after surgery, the patients’ Berg Balance scores and Tinetti Gait and Balance scores were increased by 3.60±5.00% and 4.14±6.55%, respectively. Postoperative increase in visual acuity was significantly greater in the 16 patients with visual acuity less than 0.05 (>2 LogMAR) (p=0.036), but balance scores were not significantly different.

**Conclusion:**

Visual acuity is significantly improved one month after cataract surgery, which also leads to significant increases in low functional balance scores among patients with poorer vision. The rapid increase in vision after cataract surgery enhances balance skills, resulting in safer mobility and increased quality of life.

## Introduction

Cataract is a treatable condition that generally emerges in old age and is a leading cause of vision loss. Today, increases in education level and the average human lifespan are increasing the demand for cataract surgery. In addition to reduced vision, cataracts also cause visual problems such as glare, defects in color vision, and loss of contrast sensitivity and depth perception. These symptoms lead to problems such as loss of balance, less independent mobility, falls, injuries, and increased mortality risk in individuals with visual impairment.^1^ Every year, approximately 646,000 people worldwide lose their lives due to falls, and according to a report from the World Health Organization, falls are the second most common cause of injury-related deaths.^2^ Furthermore, the daily activities of elderly patients are affected and patient’s quality of life is impaired.^3^ Cataract surgery is now performed not only to treat blindness, but to improve quality of life. Atasavun and Akı^4^ reported that studies in different age groups have shown that the incidence of falls is higher among the visually impaired than among individuals with auditory impairment or with no visual impairment. According to 2016 data from the Australian Institute of Health and Welfare, falls become a common problem over the age of 65 and are one of the leading causes of accidental deaths (40% for men and 66% for women).^5^ Other studies have shown that an average of 30% of older adults fall once a year and 20% of them are hospitalized as a result of these falls.^6,7^

Although there are various studies in the literature evaluating the relationship between vision and balance, some of these studies have not demonstrated functional balance, while vision was not evaluated objectively in others.^8^ The relationship between vision and balance cannot be fully elucidated without an objective assessment of vision level, especially for patients with low vision.^9^ Some studies involved retrospective evaluations of surveys conducted in patients who had history of falls. However, various factors may be overlooked in these studies due to inaccurate recollection of events. In addition, because balance is affected by many parameters such as age, sex, muscle strength, vestibular function, medication use, and comorbidities, it is difficult to form a well-matched control group and establish a direct relationship between vision and balance.^10^ Therefore, in the present study, we prospectively enrolled a group of patients whose characteristics did not differ except for vision. By evaluating these patients before and after cataract surgery, the relationship between vision and balance was revealed more clearly, without confounding by other variables.

In this study, we investigated the effect of vision increase in adult cataract patients after phacoemulsification surgery and intraocular lens implantation on functional balance skills.

## Materials and Methods

Adult patients with cataract who were recommended phacoemulsification and intraocular lens implantation in our center between May and October 2016 were enrolled in the study. The study was designed in accordance with Declaration of Helsinki criteria and each participant signed an informed consent form before the study. The study was approved by the Antalya Training and Research Hospital ethics committee.

Exclusion criteria for the study were presence of chronic diseases such as rheumatoid arthritis or osteoarthritis, immobility with or without assistive devices or severe lower extremity deformities that might affect mobility, vestibular problems, history of stroke, and presence of dementia or memory problems.

Demographic data such as age, sex, marital status, education level, and occupation were determined for the individuals who met the study criteria and agreed to participate in the study. The patients’ corrected visual acuity was assessed using Snellen E chart before and after cataract surgery. Functional balance was evaluated by the same physical therapist before and one month after surgery using the Berg Balance Scale (BBS) and Tinetti Gait Test (TGT) and Tinetti Balance Test (TBT).^11,12^

**Figure 1:**
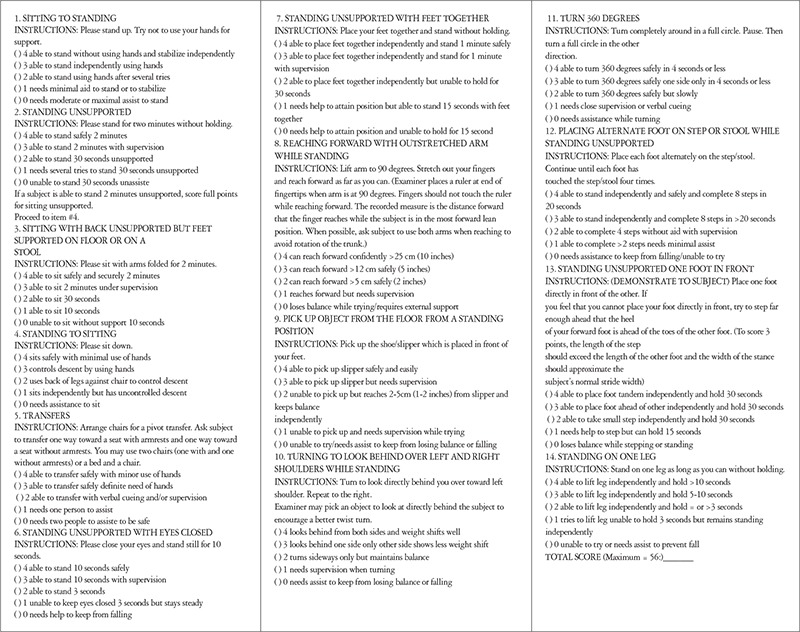
Berg Balance Scale

**Berg Balance Scale:** Designed primarily to assess balance and determine risk of falls in older adults, the BBS consists of 14 items for direct observation of performance. A ruler, stopwatch, chair, step, an area that allows 360 degrees of rotation, and 15-20 minutes are needed to perform the BBS. Each item is scored 0-4 according to the patient’s ability to meet the time and distance requirements of the test. A score of 4 indicates ability to complete the task independently. The maximum score is 56. A score of 0-20 is interpreted as poor balance, 21-40 as acceptable balance, and 41-56 as good balance (Figure 1).

**Figure 2:**
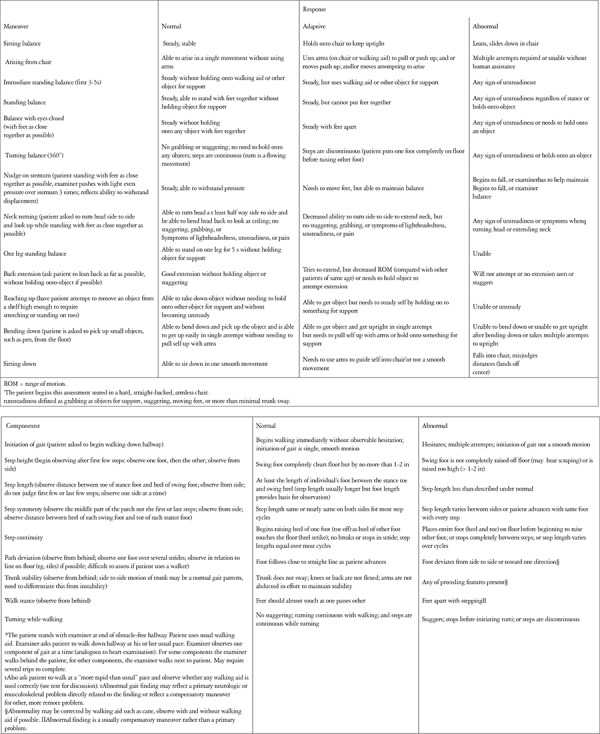
Tinetti Balance and Gait Tests

**Tinetti Gait and Balance Tests:** This test is preferred for determining the risk of falls, especially in the elderly, and consists of 13 items for balance and 9 items for gait. Items are scored binarily (0 or 1) or on a 3-point scale (0-2). Scores are calculated over a maximum of 16 for balance and 12 for gate, for a maximum total score (gait + balance) of 28 (Figure 2).

### Statistical Analysis

The research data were entered into a spreadsheet file and evaluated with Microsoft^®^ Excel^®^ for Mac 2011 version 14.5.9 (151119) and Statistical Package for the Social Sciences version 20 (SPSS 20) (IBM, New York, USA) software. Female and male patients were compared in terms of age, visual acuity, and balance using Mann-Whitney U test. Relationships between the parameters of age, visual acuity, and balance were evaluated with Pearson correlation analysis. Balance scores before and after cataract surgery were compared using dependent-samples t-test. Patients with high (<2 LogMAR) and low (>2 LogMAR) preoperative visual acuity were compared using independent-samples t-test. Values associated with balance were analyzed with one-way ANOVA. P values less than 0.05 were considered statistically significant.

## Results

This prospective study included a total of 51 patients, 27 (52%) women and 24 (48%) men, who met the inclusion criteria. Their mean age was 66.96 (33-87 years). There were no significant differences between the male and female patients in terms of age or preoperative and postoperative visual acuity (Table 1). Mean preoperative visual acuity was 1.32±0.75 (0.3-2.5) LogMAR. Visual acuity increased significantly in both groups postoperatively (p<0.001).

Both male and female patients also showed significant postoperative improvements in balance. At postoperative 1 month, BBS scores were increased by 3.60±5.00% (0-20%), while TGT and TBT were increased by 4.14±6.55% (0-38.46%). The increase in TGT and TBT scores was found to be statistically significant (Table 2).

Comparison based on preoperative visual acuity revealed a significantly greater increase in postoperative 1-month visual acuity among the 16 patients in the >2 LogMAR (<0.05) group compared to the 35 patients in the ≤2 LogMAR (≥0.05) group (p=0.036). However, there was no significant difference between these two groups in terms of increase in balance and gait scores (Table 3).

## Discussion

Vision is one of the most important factors in maintaining balance and preventing falls. Kulmala et al.^13^ demonstrated in their study of elderly women that visual impairment had the greatest impact on falls when compared with other sensory impairments. This finding was attributed to the fact that other senses can somewhat compensate for deficiencies by filling in gaps regarding posture and balance. In a study performed in Turkey, it was shown that individuals with visual impairments accounted for a significantly higher proportion of a group of adults with fall-related extremity fractures (78.6%) compared to the control group (38.1%).^14^ This led the authors to conclude that first assessing vision and treating any detected impairments is imperative for the prevention of falls and accidents. These studies demonstrate that the incidence of fall-related fractures can be reduced through regular eye examination in adults, regular use of eyeglasses among patients with refractive error, and timely interventions for treatable eye disorders, primarily cataracts. A study of 1361 individuals in China also indicated that corrected visual acuity lower than 0.5 in the better-seeing eye significantly increased the incidence of falls.^15^

In the present study, all patients exhibited significant visual improvement at 1-month follow-up after cataract surgery, consistent with the literature.^16^ In addition to postoperative increase in vision, our patients also had higher balance scores on the BBS and TBT. This suggests that the risk of falls will decrease as a result of higher balance scores associated with improved vision.

According to the results obtained from all of the balance tests used, we observed that the women had lower balance scores than the men in our study. To et al.^17^ also reported that the incidence of falls was three times higher in females than males in their 2014 study. However, when we analyzed postoperative changes in balance scores, we found that balance scores increased more among the women in our study. The increase in TBT scores was statistically significantly in females (p=0.003) but not in males. This may indicate a stronger association between balance and vision in women.

Preoperative vision level also affects the benefit of cataract surgery on visual outcome.^18^ When we compared our patients’ results in two groups based on preoperative visual acuity level, the group with preoperative visual acuity worse than 0.05 showed a significantly larger increase in postoperative 1-month visual acuity than the other group (p=0.036). However, we detected no significant differences between these two groups in terms of increases in balance or gait scores. Although studies evaluating the effect of vision on balance and falls have yielded very different results, most authors agree that increased vision has a positive impact on the ability to maintain balance.^15,17,19,20^ In contrast to these data, the authors of a study published in 2015 argued that visual impairment in elderly cataract patients was not associated with balance disorders or falls.^21^ Furthermore, Cumming et al.^22^ found that improving older adults’ vision through treatment actually increased the incidence of falls, but they attributed this discrepant result to the fact that the patients became more mobile and active when their vision was restored. In their study of 413 patients over 50 years old, To et al.^17^ observed a 78% reduction in risk of falls after surgery on the first eye and 83% after surgery of the second eye. Foss et al.^23^ reported that the incidence of falls decreased by 32% after surgery on the second eye. Desapriya et al.^19^ showed that early cataract surgery substantially improved visual acuity but had no significant effect on falls. However, Supuk et al.^24^ emphasized that after cataract surgery, there was a significant decrease in vertigo rather than in the incidence of falls.

Most of the published studies on this topic have been retrospective, with patients’ visual acuities analyzed after examining the patients’ records or conducting surveys regarding their falls history.^4,14,15,16,17,24,25,27^ Compared to objective tests, these surveys both provide inadequate information and may give rise to misleading data due to patients’ inaccurate recollection of past events. Moreover, as visual acuity is measured at the time of the study, accurate information cannot be obtained about the patients’ visual acuity at the time of falling. The scientific significance of our study lies in the fact that it was planned as a prospective study and the patients were tested and evaluated at the same time by an ophtalmologist and a physical therapist. Most previous studies focused on vision and falls incidence, but there are few studies that have tested and compared patients’ pre- and postoperative balance.

Like many other studies, the current study demonstrates that, by referring individuals to eye examinations at regular intervals, quality of life can be increased and a substantial proportion of falls can be prevented in older adults.^3,14,15,17,20,28,29^

### Study Limitations

One limitation of our study is that vision level varied in the patients’ fellow eyes. While the fellow eye also had cataract in some patients, others had near perfect vision (mean LogMAR=0.48). This might have affected their balance scores. The visual benefit of cataract surgery might also vary depending on the status of the fellow eye.^18^ Moreover, sudden increase in vision in one eye while the other eye still has cataract may cause imbalanced vision and consequently impaired balance rather than improved balance. In fact, Meuleners et al.^25^ found that the incidence of falls requiring hospitalization doubled in the interval between first and second cataract surgeries compared with the preoperative period, and argued that ophthalmologists must warn patients to be more careful regarding falls after the first surgery.

Another limitation of the study is that we did not assess any other vision functions such as visual field, contrast sensitivity, depth perception, or color vision, factors that may also play a role in increasing the risk of falls. However, it is known that most of these parameters also improve after cataract surgery.^3,26^ Therefore, we believe that the cataract surgery we performed corrected these parameters to some degree along with visual acuity.

## Conclusion

This study demonstrates that phacoemulsification and intraocular lens implantation significantly increases visual acuity within the first postoperative month. As a result, the low functional balance scores of individuals with severe visual impairment increased significantly. This significant postoperative improvement in vision functions may contribute to better balance and enhance patients’ quality of life.

## Figures and Tables

**Table 1:**
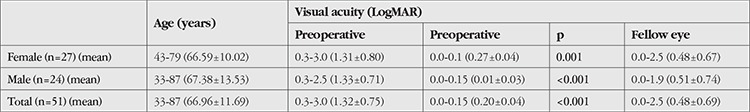
Demographic characteristics of the study patients and their visual acuity levels before and after cataract surgery

**Table 2:**
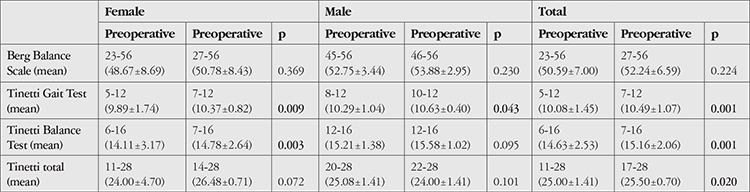
Balance scores of the study patients before and after cataract surgery

**Table 3:**
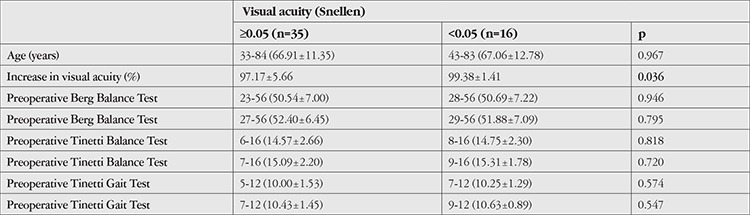
Changes in balance scores according to visual acuity before cataract surgery (Mean values are given in parentheses)
